# Supplementary material for: Effect of Lentinula edodes on Morphological and Biochemical Blood Parameters of Horses
Source: Animals (Basel). 2022 Apr 25;12(9):1106. doi: 10.3390/ani12091106 (PMC9100334; doi:10.3390/ani12091106)
Supplement: Supplementary file 1 [file animals-12-01106-s001.zip › animals-1657727-supplementary.pdf]

Suplementary Material S1

Table S1. Median of blood morphological parameters for the supplemented (G1) and control group (G0).

Parameter, unit	Median									
	Session 1 (S1)		Session 2 (S2)		Session 3 (S3)		Session 4 (S4)		Session 5 (S5)	
	G1	G0	G1	G0	G1	G0	G1	G0	G1	G0
WBC, 10^9/L	8.45	8.245	7.94	7.47	7.76	8.23	6.78	7.31	7.43	7.41
NEU, 10^9/L	4.95	4.97	4.36	3.92	4.85	5.07	4.26	4.2	4.42	3.73
NEU%	61.2	61.7	62.8	57.35	64.5	61.2	60.3	57.5	58	55.45
LYM, 10^9/L	2.09	2.42	2.09	2.63	1.94	2.43	2.09	2.27	2.39	2.51
LYM%	26.3	28.5	27.5	32.8	25.1	31.55	31.7	34	32.2	35.5
MONO, 10^9/L	0.48	0.4	0.4	0.4	0.47	0.38	0.38	0.33	0.41	0.38
MONO%	5.7	4.95	5.4	5.25	5.9	5	5.6	4.6	5.7	5.55
EOS, 10^9/L	0.24	0.26	0.3	0.32	0.27	0.19	0.21	0.19	0.17	0.17
EOS%	3.3	2.95	3.9	3.8	3.6	2.4	2.7	2.9	2.8	2.05
BASO, 10^9/L	0.08	0.05	0.07	0.04	0.06	0.04	0.07	0.05	0.06	0.04
BASO%	1	0.6	0.9	0.6	0.9	0.5	1	0.75	1.2	0.6
RBC, 10^{12}/L	6.47	6.82	7.04	7.29	7.13	7.17	7.28	6.93	7.27	7.36
HGB, 10^{12}/L	111	108	114	122.5	116	115	119	112	122	114.5
HCT, L/L	0.323	0.312	0.332	0.341	0.327	0.328	0.343	0.324	0.352	0.3285
MCV, fL	46.4	46.35	47.3	46.45	46.7	45.6	47.6	46.55	47.4	46.9
MCH, mmol/I	2.09	16.2	2.09	16.25	1.94	16.1	2.09	16.1	2.39	16.15
MCHC, g/l	354	354	346	351	354	352	345	345.5	351	347.5
PLT, 10^{12}/L	115	106	118	126	136	121	116	117	115	107

Table S2. Median of blood biochemical parameters for the supplemented (G1) and control group (G0).

Parameter, unit	Median									
	Session 1 (S1)		Session 2 (S2)		Session 3 (S3)		Session 4 (S4)		Session 5 (S5)	
	G1	G0	G1	G0	G1	G0	G1	G0	G1	G0
Albumin, g/L	31.7	29.9	32.8	31.5	34.8	32.8	33.7	32.0	33.0	31.8
AP, U/L	209.0	192.5	237.0	189.5	199.0	156.0	195.0	154.5	210.0	147.5
AST, U/L	368.7	338.1	356.2	355.2	359.2	330.5	380.1	359.5	402.7	342.1
Total protein, g/L	67.2	64.0	67.0	65.4	69.8	65.9	66.6	63.3	62.2	60.2
Bilirubin, µmol/L	20.1	19.9	20.3	20.9	24.4	20.2	19.2	21.3	16.1	16.9
Chlorides, mmol/L	100.8	102.2	100.6	101.1	99.5	100.8	99.4	100.9	98.4	99.4
Cholesterol, mmol/L	2.0	2.0	1.8	2.0	1.8	1.9	1.9	2.1	1.9	2.1
CK, U/L	256.0	237.5	376.0	290.5	371.0	359.5	322.0	300.0	331.0	265.0
P, mmol/L	0.9	1.0	1.2	1.2	1.0	1.1	1.0	1.1	0.9	1.0
GLDH, U/L	3.3	3.2	2.8	2.2	3.1	3.3	3.1	3.1	3.1	3.0
Glucose, mmol/L	5.2	5.0	4.7	4.8	4.6	4.5	4.8	5.0	4.8	4.9
GGTP, U/L	12.6	10.7	12.4	10.0	13.2	11.6	13.4	11.1	14.6	11.5
Creatinine, µmol/L	94.4	87.3	103.7	96.4	101.6	91.9	104.9	99.4	98.9	100.6
LDH, U/L	354.2	404.7	361.0	372.9	436.4	417.8	342.1	375.5	318.9	315.9
Mg, mmol/L	0.9	0.9	0.8	0.8	0.8	0.7	0.9	0.8	0.8	0.8
Urea, mmol/L	6.6	7.6	5.7	6.1	7.0	7.1	6.1	6.1	6.2	6.6
K, mmol/L	4.0	3.9	4.2	4.2	3.5	3.6	3.8	4.0	3.8	3.9
Na, mmol/L	136.5	139.2	138.5	139.4	138.9	139.3	137.1	137.4	136.1	136.2
TGL, mmol/L	0.200	0.280	0.160	0.185	0.190	0.205	0.170	0.195	0.220	0.265
Ca, mmol/L	3.1	3.0	3.1	3.0	3.1	3.1	3.2	3.0	3.1	3.0
Globulins, g/L	34.0	33.9	33.4	33.4	36.3	33.3	34.4	30.5	31.0	28.4
Albumin/globulin ratio, mmol/L	0.95	0.88	0.97	0.96	0.96	1.09	1.05	1.03	1.10	1.11
Lactic acid, mmol/L	0.69	0.69	0.98	1.02	1.21	1.09	0.84	1.01	0.81	0.88
